# *TkJAZs-TkMYC2-TkSRPP/REF* Regulates the Biosynthesis of Natural Rubber in *Taraxacum kok-saghyz*

**DOI:** 10.3390/plants13152034

**Published:** 2024-07-24

**Authors:** Yulin Wu, Gaoquan Dong, Fengqi Luo, Hao Xie, Xiaodong Li, Jie Yan

**Affiliations:** Key Laboratory of Xinjiang Phytomedicine Resource and Utilization of Ministry of Education, Xinjiang Production and Construction Corps Key Laboratory of Oasis Town and Mountain-Basin System Ecology, College of Life Sciences, Shihezi University, Shihezi 832003, China; yulinwu2023@163.com (Y.W.); donggq2023@163.com (G.D.); fengqiluo2025@163.com (F.L.); haoxie0526@163.com (H.X.); xiaodongli202407@163.com (X.L.)

**Keywords:** *Taraxacum kok-saghyz* Rodin, *JAZ* and *MYC2* genes, overexpression, rubber-producing plant

## Abstract

*Taraxacum kok-saghyz* (TKS) is a natural rubber (NR)-producing plant and a model plant for studying the biosynthesis of NR. Analyzing and studying the biosynthetic mechanism of NR is an important way to cultivate high-yield rubber TKS varieties. JAZ proteins, which belong to the Jasmonate ZIM domain family, function as negative regulators in the jasmonic acid (JA) signal transduction pathway. MYC2 is typically regarded as a regulatory factor for the target genes of JAZ proteins; JAZ proteins indirectly influence the gene expression regulated by MYC2 by modulating its activity. Theoretically, JAZ is expected to participate in growth, development, and responses to environmental cues related to rubber and biomass accumulation in TKS, all of which rely on the interaction between JAZ and MYC2. In this study, we identified 11 TkJAZs through homology searching of the TKS genomes and bioinformatics analyses. Subcellular localization, Y2H, and BiFC analysis demonstrate that TkJAZs and TkMYC2 are localized in the nucleus, with all TkJAZs and TkMYC2 showing nuclear colocalization interactions. Overexpression of *TkMYC2* in TKS inhibited leaf development, promoted root growth, and simultaneously increased NR production. RNA-seq and qRT-PCR analysis revealed that the *TkSRPP/REF* genes exhibit varying degrees of upregulation compared to the wild type, upregulating the *TkREF1* gene by 3.7-fold, suggesting that *TkMYC2* regulates the synthesis of NR by modulating the *TkSRPP/REF* genes.

## 1. Introduction

The Russian dandelion, also known as the rubber dandelion, or *Taraxacum kok-saghyz* (TKS), is a perennial herbaceous dicotyledonous plant belonging to the Asteraceae family [1]. Early global studies have determined that the primary source of natural rubber (NR:cis-1,4-polyisoprene) production in rubber plants is their roots. In particular, the NR content in the root dry weight can range from 2.89% to 27.89% [1,2,3]. TKS provides greater benefits in agricultural production than *Hevea brasiliensis* because it has a shorter harvest cycle, can be seeded right away, is very adaptable, and can be harvested mechanically [4,5,6].

Plants use jasmonic acid (JA) as a phytohormone to regulate a variety of growth and developmental processes, including blooming, leaf senescence, root growth, and more [7]. Second, it has been discovered that JA stimulates the expression of various important secondary metabolites and proteins, such as some anti-insect proteinase inhibitors, amino acid derivatives, terpenes, phenylpropanoids, alkaloids, and anti-nutritional proteins [8,9,10,11,12,13]. Furthermore, the JA signaling pathway aids in the formation of defense-related morphological features like nectaries, resin tubes, and glandular hairs, which either directly or indirectly contribute to plant defense by producing a variety of chemical compounds [14,15,16,17,18,19]. JA must undergo enzymatic conjugation with isoleucine (Ile) to activate plant defense. The resulting JA-Ile conjugates bind to and activate a ubiquitin ligase complex (SCF^COI1^), which promotes the degradation of jasmonate ZIM domain (JAZ) proteins [20]. JAZ protein is localized in the nucleus of the cell and is a zinc finger structural protein characterized by the presence of TIFY and Jas (also known as CCT2) domains [21]. The TIF[F/Y] XG conserved sequence, located in the TIFY domain at the N-terminal region, can bind to downstream MYC2 [22]. The concentration of JA-Ile in cells is minimal in plants growing normally. As a major transcriptional suppressor during this period, the JAZ proteins interact with particular transcription factors, such as MYC2, through its ZIM domain to stop them from increasing the expression of JA-sensitive genes [23,24]. A cell’s levels of JA-Ile rise sharply in response to stressors including insect infestation, mechanical harm, or pathogen invasion. These JA-Ile conjugates subsequently enter the nucleus via specific transport proteins, thereby emitting signals to activate the JA signaling pathway. Elevated JA-Ile levels in the nucleus facilitate its binding to the F-box protein COI1 within the SCF^COI1^ complex, forming the COI1-JAZ co-receptor complex [24]. This interaction relieves the inhibition of JA-responsive gene expression, with MYC2 being a key regulator, through ubiquitination of the JAZ protein and its subsequent degradation by the 26S proteasome [25].

MYC2 is a transcription factor belonging to the basic helix loop helix (bHLH) family. It has an alkaline area called the Pfam bHLH-MYC-N conserved domain, which comprises a bHLH conserved domain and two short-conserved domains, JID and TAD. The conservative domain of bHLH is placed at the C-end and largely carries out a catalytic function; the conserved domain of Pfam bHLH-MYC-N is located at the N-terminus and exerts a DNA binding function [26,27,28]. MYC2 is involved in several biological activities. It has been demonstrated that MYC2 reduces the growth of Arabidopsis roots in experiments involving both overexpression and mutations of the MYC2 gene in Arabidopsis [29]. Subsequent investigation revealed that MYC2 in the JA signaling pathway directly targets and binds to the G-BOX element of the *ERF115* promoter to regulate the expression of the stress response gene *ERF115*; the expression of genes related to the RBR-SCR molecular network, which *ERF115* regulates, activates the activity of root stem cell tissue centers collectively. *ERF109*, which is induced by JA signaling and is located upstream of *ERF115*, can also activate the expression of the *CYCD6* gene and promotes root stem cell tissue regeneration [30].

On the surface of rubber particle membranes, small rubber particle protein (SRPP) is present [31,32,33]. It forms subunits of rubber transferase (RT-ase) complexes, which play a crucial role in rubber chain elongation and determining the quality of NR [34,35]. Rubber content in roots may be increased by overexpressing *TkSRPP3* in TKS, and rubber content and the molecular size of rubber hydrocarbons in the root were reduced as a result of RNA interference targeting the expression of the *TkSRPP3* gene [34]. In addition to the aforementioned studies, the reduction in *Taraxacum brevicorniculatum TbSRPPs* expression through RNAi technology can impact the stability of rubber particles and decrease the dry rubber content by 40–50% [36]. This indicates that *SRPP* family genes are pivotal in NR biosynthesis; SRPP is also impacted by the JA signaling pathway, and studies have revealed that the mechanism of JA regulating NR synthesis is exogenous JA inducing COI1 to bind to JAZ3 protein, releasing MYC2 from the MYC2-JAZ3 complex and binding to *SRPP1* and *FPS1* promoters, thereby regulating rubber synthesis [37].

This study provides a comprehensive identification and characterization of the JAZ gene family in TKS. We analyzed the phylogeny, conserved domains, motifs, gene structures, chromosomal localization, and collinearity of the identified TkJAZ genes. Subsequently, we investigated the transcriptional levels of these *TkJAZs* in TKS by means of MeJA-RNA-seq (extract RNA from the roots of TKS treated with MeJA for transcriptome sequencing) data analysis. Next, the subcellular localization, Y2H, and BiFC experiment confirmed that *TkMYC2* is regulated by all *TkJAZs* and determined *TkMYC2* as the crucial research gene. Finally, we overexpressed the *TkMYC2* gene in TKS and measured the physiological indicators of the overexpression of *TkMYC2* type (OE-*TkMYC2*) TKS, including NR yield. Meanwhile, we selected the NR biosynthesis-associated gene family *TkSRPP/REF* for analysis and investigated the transcriptional levels of *TkSRPP/REF* in OE-*TkMYC2* TKS by qRT-PCR analysis. The results obtained will contribute to a deeper understanding of the roles of *TkJAZs* and *TkMYC2* in the JA signaling pathway in relation to NR production in TKS roots.

## 2. Results

### 2.1. Identification and Conserved Domains and Gene Structure Analysis of the TkJAZ Gene Family

Two approaches were used to cooperatively identify *TkJAZ* gene family members. Through a local BLAST analysis using the protein sequences of the *Arabidopsis* JAZ family as the query and a database search for the TIFY/JAS domain, a total of 11 *JAZ* genes were found in the TKS genome (Table S1) assembly established in the whole genome data of TKS published in 2017 [38].

**Figure 1:**
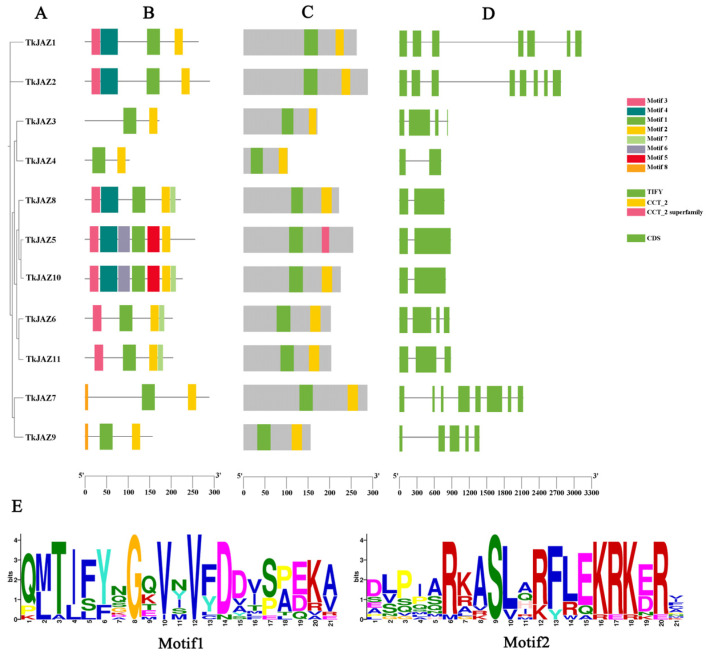
Phylogenetic relationship, motif composition, TIFY/JAS(CCT-2) conserved domains, and gene structure of the TkJAZ family proteins. (**A**) The phylogenetic tree of the TkJAZ family members. (**B**) The motif composition in TkJAZs. Different colored boxes represent putative motifs. (**C**) TIFY/JAS(CCT-2) domain of TkJAZs. (**D**) Exon/intron structure of the *TkJAZ* genes. The green boxes represent exons and the black lines introns. (**E**) The abscissa of sequence logos refers to the amino acid with the highest frequency and the ordinate represents the relative frequency of the corresponding amino acid.

Phylogenetic tree analysis within the JAZ family (Figure 1A) revealed that the 11 TkJAZ proteins were divided into six subgroups. The conserved motifs and domains of the TkJAZ proteins were identified using the MEME motif search engine and the CD-Search structural domain prediction program (Figure 1B,C). The exon–intron organization of the TkJAZ genes was analyzed using the Gene Structure Display Server tool (Figure 1D). Among the 11 TkJAZs, a total of eight motifs were identified (Figure 1B). As predicted, each TkJAZ contained two TIFY/JAS domains (Figure 1C). These TIFY/JAS(CCT-2) domains, characteristic of JAZ proteins, are present in all TkJAZs and correspond to Motif1 and Motif2 in Figure 1B. The detailed amino acid composition of these domains is shown in Figure 1E. Phylogenetic tree analysis within the JAZ family (Figure 1A) indicated that, with a few exceptions, TkJAZs within the same clade generally exhibited similar exon–intron structures (Figure 1D) and shared motif compositions (Figure 1B). This suggests that JAZ proteins within the same group may have similar biological functions.

These *TkJAZs* were given names based on chromosome numbering order and listed the number of amino acids (Table 1). Subcellular localization prediction was conducted, and the results showed that all TkJAZ proteins were localized in the nucleus.

### 2.2. Phylogenetic Analysis of the TkJAZ Gene Family

To reveal the evolutionary relationship of the *JAZ* gene family in plants, a phylogenetic tree was created using the deduced protein sequences from TKS and orthologous proteins from three types of herbaceous plant genomes used in this study: *Arabidopsis thaliana* (13 AtJAZ), *Oryza sativa* (15 OsJAZ), and *Hevea brasiliensis* (18 HbJAZ).

**Figure 2:**
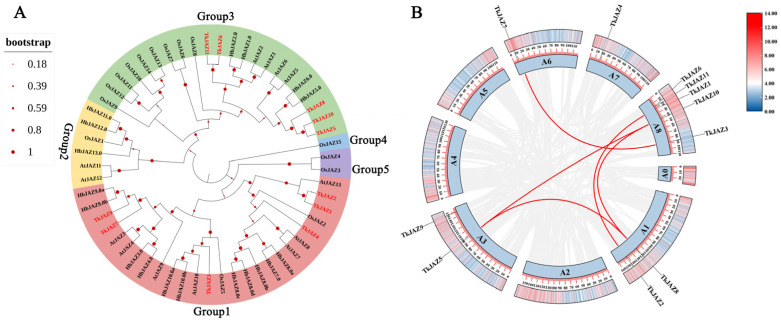
Phylogenetic tree, chromosomal localization, and collinearity of *TkJAZ* genes in the TKS genome. (**A**) Phylogenetic tree of JAZs from *Arabidopsis thaliana*, *Oryza sativa*, *Hevea brasiliensis,* and *Taraxacum kok-saghyz*. The colors represent the five JAZ groups: red, group1; yellow, group2; green, group3; blue, group4; and purple, group5. (**B**) Chromosomal localization and collinearity of the *TkJAZ* genes.

Five unique groups (Groups 1, 2, 3, 4, and 5) could be formed using the JAZs from the four species listed above (Figure 2A). Notably, the majority of TkJAZs and HbJAZs share a subfamily and are closely related genetically. This suggests that the JAZ proteins of TKS and the rubber tree may possess similar functional structures with evolutionary relationships. These proteins likely play roles in regulating the genes associated with NR biosynthesis.

### 2.3. Chromosomal Distribution and Collinearity of the TkJAZs

To perform chromosome localization and collinearity analysis, we aligned 11 TkJAZ genes to the updated TKS genome data from 2022 [39], as the 2017 TKS genome data lacked sufficient chromosomal information. Based on the results, it was determined that the 11 *TkJAZ* genes are spread among five TKS chromosomes: 2 genes are located on A1 and A3, 1 gene is located on A6 and A7, and 5 genes are located on A8 (Figure 2B). In addition, collinearity analysis revealed that certain *TkJAZ* genes located on the same or different chromosomes showed collinearity. For instance, *TkJAZ1* and *TkJAZ2*, *TkJAZ5* and a two-gene cluster (*TkJAZ8* and *TkJAZ10*), and *TkJAZ3* and *TkJAZ7* displayed collinearity (Figure 2B). These findings suggest that the expansion of the *JAZ* gene family in TKS likely arose from both whole and small-scale genome duplications, as well as tandem duplications. Furthermore, *TkJAZ* genes exhibiting collinearity may share more similar biological functions.

### 2.4. Protein–Protein Interaction Prediction and RNA-Seq Dataset Analysis of TkJAZs

**Figure 3:**
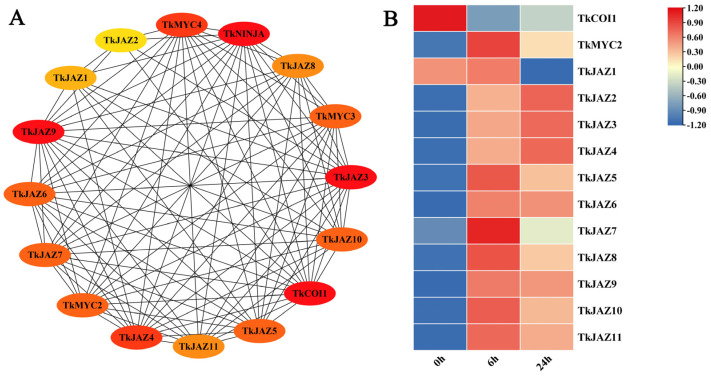
Protein interaction prediction and expressional patterns of the *TkJAZ* gene family. (**A**) Protein interaction network diagram. (**B**) Hierarchical clustering and heat map representation of *TkJAZs* expression based on the MeJA-RNA-seq data. The data are the average of three replicates.

The STRING protein interaction prediction tool was used to evaluate the interaction relationships between these TkJAZs and related proteins in JA signaling pathways (Figure 3A), and the expression patterns of 11 *TkJAZs* in TKS were evaluated using the RNA-seq dataset processed by MeJA (Figure 3B).

According to predictions of protein interactions, TkJAZs interact with TkCOI1 and TkMYC2 in all cases (Figure 3A). Following a 6 h MeJA treatment, RNA-seq data analysis revealed that *TkJAZs* and *TkMYC2* expression levels rose whereas *TkCOI1* expression levels fell. Following a 24 h MeJA treatment, most *TkJAZs* (*TkJAZ1*, *TKJAZ5*-*TkJAZ11*) and *TkMYC2* showed decreased expression levels, but *TkCOI1*, *TkJAZ2*, *TkJAZ3*, and *TkJAZ4* showed higher expression levels (Figure 3B). This suggests that MeJA treatment may activate the binding of TkCOI1 to TkJAZs, leading to a decrease in *TkCOI1* expression, while MeJA treatment can also activate the expression of *TkJAZs*, and the consumption of *TkJAZs* increases the expression of *TkMYC2*. This analysis helped to explain the role of *TkJAZs* in the JA signaling pathway.

### 2.5. Subcellular Localization Analysis

**Figure 4:**
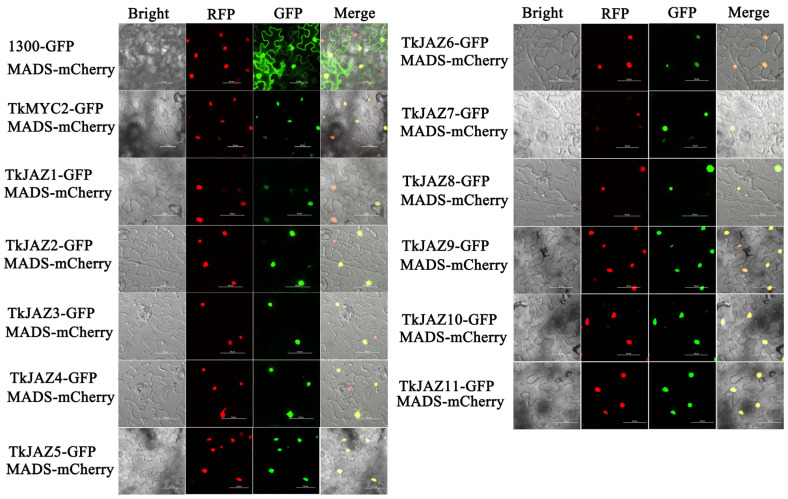
Subcellular localization of 11 *TkJAZ* genes and *TkMYC2*. Colocalization of *TkJAZ1*-GFP~*TkJAZ11*-GFP and *TkMYC2*-GFP with nuclei positioning marker *MADS*-mCherry in *N. benthamiana* leaves. GFP: 488 nm; RFP: 561 nm; scale bars = 50 μm.

To determine the subcellular localization of *TkJAZs* and *TkMYC2*, expressing vectors of green fluorescent protein (GFP)-fused TkJAZs and TkMYC2 proteins were constructed and co-transfected, respectively, with the nuclei positioning marker protein *MADS*-mCherry into *N. benthamiana* leaves. As depicted in Figure 4, the *MADS*-mCherry protein was located in the nucleus as anticipated. The merged images reveal a high overlap of GFP and RFP fluorescence signals, confirming that TkJAZs and TkMYC2, along with the nuclear localization marker protein *MADS*-mCherry, are also localized in the nucleus.

### 2.6. Y2H and BiFC Analysis: The Important Value of TkMYC2 in TKS

**Figure 5:**
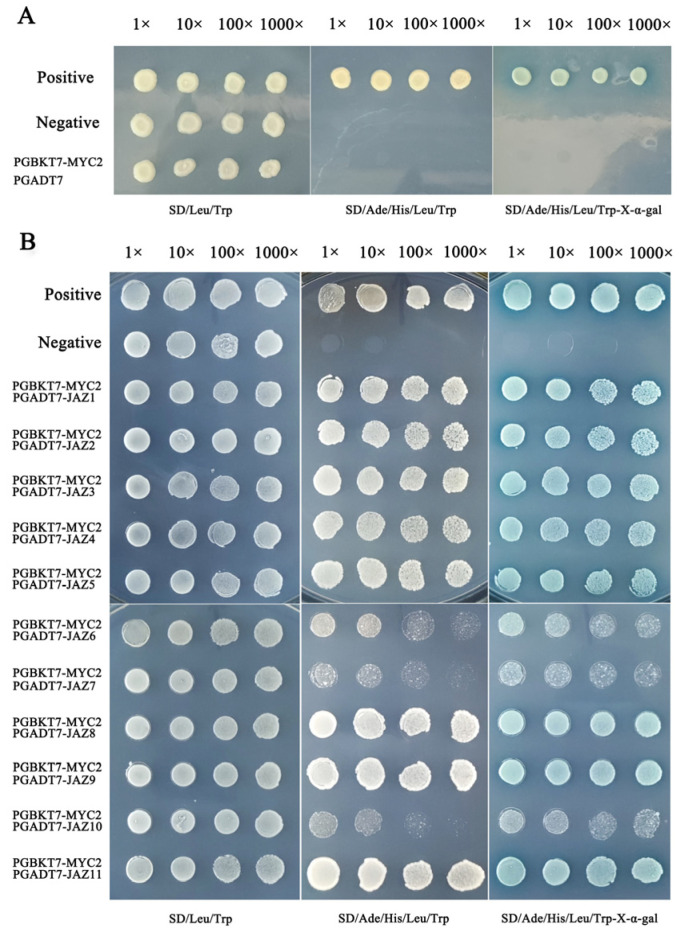
Y2H of TkJAZs and TkMYC2 proteins. (**A**) Verification of automatic activation of bait yeast. (**B**) Verification of point-to-point interactions between TkJAZs and TkMYC2. Positive: pGBKT7 − p53+pGADT7 − T, Negative: pGBKT7 − lam+pGADT7 − T.

As the first step in any Y2H determination, it is necessary to confirm that the bait will not appear automatically to activate reporter genes without a prey protein. At this point, the full-length *TkMYC2* is fused with the pGBKT7 vector to construct a bait vector, and then the pGADT7 empty vector and pGBKT7-*TkMYC2* vector are co-transformed into yeast receptive cells Y2HGold to test their own activation. As shown in Figure 5A, the yeast cells that transformed the bait vectors into fused full-length *TkMYC2* did not exhibit autoactivation.

Using the Y2H point-to-point verification experiment to determine the interaction relationship of the TkJAZs and TkMYC2, the pGADT7-*TkJAZs* vector and pGBKT7-*TkMYC2* vector are co-transformed into yeast receptive cells Y2HGold to test their protein–protein interaction. As shown in Figure 5B, TkJAZs and TkMYC2 have extensive interactions because Y2H yeast colonies grow on SD/Ade/His/Leu/Trp-X-α-gal medium and display blue color. Among them, TkMYC2 exhibits strong interactions with TkJAZ1, TkJAZ2, TkJAZ3, TkJAZ4, TkJAZ5, TkJAZ8, TkJAZ9, and TkJAZ11, while showing weak interactions with TkJAZ6, TkJAZ7, and TkJAZ10.

**Figure 6:**
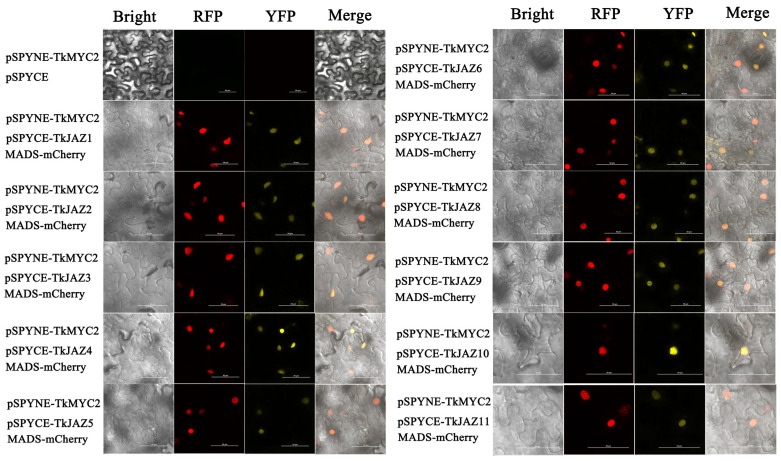
BiFC of 11 *TkJAZ* genes and *TkMYC2*. Colocalization of pSPYNE-*TkMYC2* and pSPYNE-*TkJAZs* with nuclei positioning marker *MADS*-mCherry in *N. benthamiana* leaf. YFP: 514 nm; RFP: 561 nm; scale bars = 50 μm.

To verify the interaction relationship of the TkJAZs and TkMYC2, BiFC-expressing vectors of yellow fluorescent protein (YFP)-fused TkJAZs and TkMYC2 proteins were constructed and co-transfected with the nuclei positioning maker protein *MADS*-mCherry into *N. benthamiana* leaves. As shown in Figure 6, under excitation light of 514 nm, there was a bright yellow fluorescence that converged, indicating the interaction between TkJAZs and TkMYC2. As observed in the merged images, the high overlap of YFP and RFP fluorescence signals demonstrated the TkJAZs and TkMYC2 interactions in the nucleus.

These findings suggest that TkMYC2 is crucial to the JA signaling pathway since they show that all TkJAZ proteins interact with it in the nucleus.

### 2.7. Overexpression of TkMYC2 Altered the Phenotype of TKS and Increased Natural Rubber Production

**Figure 7:**
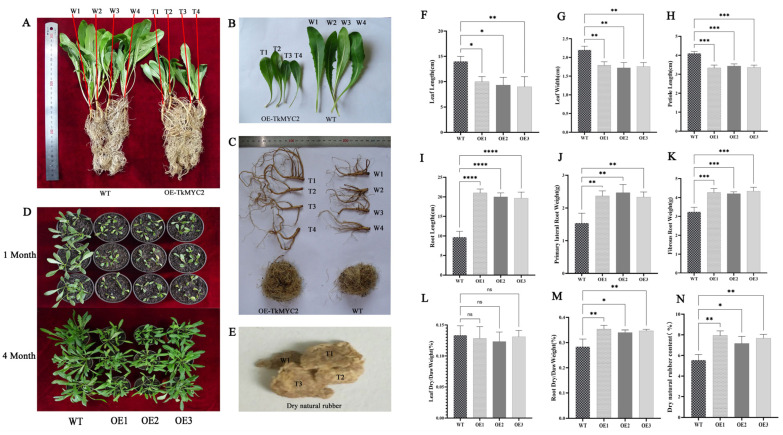
Analysis of physiological indicators between wild-type and OE-*TkMYC2* −transgenic TKS. (**A**) Comparison of overall appearance between wild-type and OE-*TkMYC2*-transgenic TKS. (**B**) Comparison of leaf size between wild-type and OE-*TkMYC2*-transgenic TKS. (**C**) Comparison of TKS roots between wild-type and OE-*TkMYC2*-type. (**D**) Comparison of growth status between wild-type and OE-*TkMYC2*-transgenic TKS at 1 and 4 months of age. (**E**) NR extracted by alkaline boiling method. (**F**–**N**) Graphical presentation of leaf length, leaf width, petiole length, root length, fibrous and lateral root weight, dry/wet leaf weight, dry/wet root weight, and dry NR content for wild-type and OE-*TkMYC2-*transgenic TKS. (T1, T2, T3, T4) represent the four biological replicates of OE-*TkMYC2*-transgenic TKS; (W1, W2, W3, W4) represent the four biological replicates of wild-type TKS. (OE1, OE2, OE3) represent the three groups of OE-*TkMYC2*-transgenic plant lines (* *p* < 0.05, ** *p* < 0.01, *** *p* < 0.0005, **** *p* < 0.0001, ns *p* > 0.05).

To investigate the effect of *TkMYC2* overexpression on NR biosynthesis, we employed the leaf disk transformation method to overexpress *TkMYC2* in TKS under the control of the 35S promoter. Three independent transgenic lines (OE1, OE2, OE3) were generated for each overexpression construct. We compared the phenotypes of OE-*TkMYC2* transgenic TKS and wild-type TKS under standard growth conditions (Figure 7). Figure 7A–C present representative images obtained during the phenotypic assessment of wild-type and OE1 transgenic lines. Figure 7D provides a bird’s-eye view, showing the overall growth of the aboveground parts of both OE-*TkMYC2* transgenic TKS and wild-type TKS. Figure 7E displays the extracted NR from both OE-*TkMYC2* transgenic TKS and wild-type TKS.

The data measurement results indicate that, compared to wild-type TKS, the OE-*TkMYC2* transgenic TKS exhibits reduced leaf sizes (Figure 7F–H), longer roots (Figure 7I), and increased biomass in both fibrous and lateral roots (Figure 7J,K). Subsequently, dehydration treatment was applied to both wild-type and OE-*TkMYC2* transgenic TKS. The results showed no significant difference in the leaf dry weight to wet weight ratio between the overexpression lines and the wild type (Figure 7L). However, the root dry weight to wet weight ratio in OE-*TkMYC2* transgenic TKS was significantly higher than that in the wild type (Figure 7M).

This suggests that the roots of OE-*TkMYC2* transgenic TKS may have synthesized more secondary metabolites, and the plant’s nutrient allocation strategy resulted in the reduction in leaf size in TKS. To further investigate, we utilized an alkaline boiling method to extract NR from the roots of six-month-old wild-type and OE-*TkMYC2* transgenic TKS, followed by weight determination. The results indicated that six-month-old OE-*TkMYC2* transgenic TKS had a higher NR content compared to the wild type (Figure 7N).

The DNA-PCR and qRT-PCR analyses for the identification of overexpressed TKS are presented in Figures S1 and S2.

### 2.8. TkMYC2 Regulates TkSRPP/REF1 Gene Expression Increase Natural Rubber Yield

**Figure 8:**
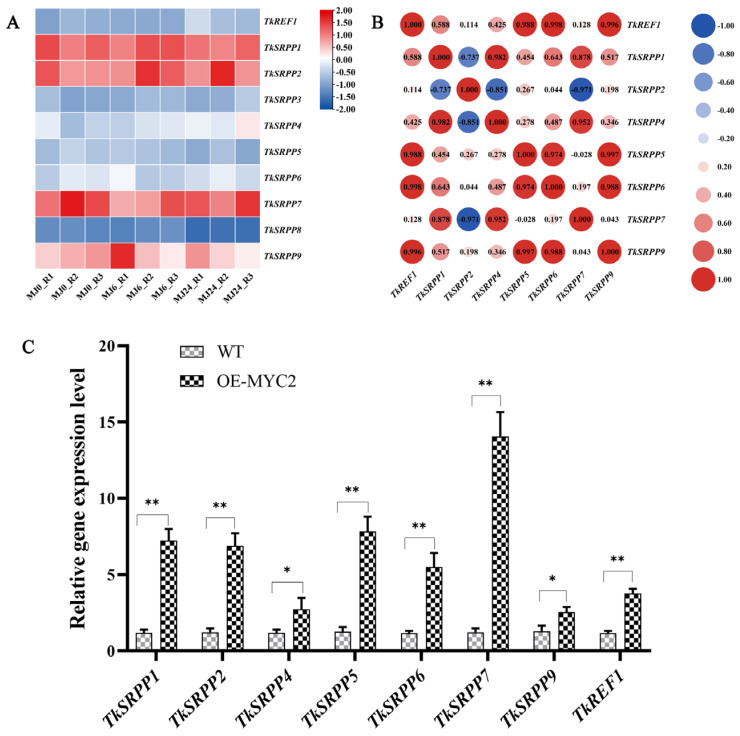
Expressional patterns of the *TkSRPP/REF* gene family. (**A**) Hierarchical clustering and heat map representation of *TkSRPP/REF* expression based on the MeJA-RNA-seq data; MJ0, MJ6, and MJ24 represent MeJA processing time; R1, R2, and R3 represent three biological replicates. (**B**) Pearson correlation coefficient analysis of the *TkSRPP/REF* genes based on the MeJA-RNA-seq data used in (**A**). (**C**) Expression levels of eight *TkSRPP/REF* genes were analyzed by qRT-PCR in OE-*TkMYC2*-transgenic TKS roots. The data represent relative expression levels normalized to those of wild-type TKS *TkSRPP/REF*, presented as means ± standard error (SE) from three independent biological replicates. (* *p* < 0.05, ** *p* < 0.01).

To elucidate the underlying reasons for the increased NR production observed in OE-*TkMYC2* transgenic TKS, we analyzed RNA-seq data focusing on the small rubber particle protein (TkSRPP) and rubber elongation factor (REF) gene family members under MeJA treatment (Figure 8A). Furthermore, their expression levels were detected by qRT-PCR in OE-*TkMYC2* transgenic TKS (Figure 8C). As shown in Figure 8A,C, the MeJA-RNA-seq data analysis revealed increased expression levels of *TkSRPP4* and *TkREF1*. In the qRT-PCR analysis, the expression levels of *TkSRPP* and *REF* genes in OE-*TkMYC2* transgenic TKS exhibited varying degrees of upregulation compared to wild-type TKS. Notably, the *TkREF1* gene was upregulated 3.7-fold in response to *TkMYC2* overexpression. These results indicate that *TkMYC2* can regulate the expression of *TkSRPP* and *REF* family genes.

Based on the aforementioned findings, *TkMYC2* may be regulated by the JA signaling pathway and can enhance the production of NR in TKS by upregulating the expression of *TkSRPP* and *REF* family genes.

Furthermore, an examination of the Pearson correlation coefficient (PCC) was performed on the *TkSRPP/REF* transcription data (Figure 8B). The findings demonstrated significant correlations between the expression levels of *TkREF1*, *TkSRPP5*, *TkSRPP6*, and *TkSRPP9* and the majority of other *TkSRPP/REF* genes. Combined with the qRT-PCR results, this suggests that *TkREF1*, *TkSRPP5*, *TkSRPP6*, and *TkSRPP9* may play more pivotal roles in NR biosynthesis and are worthy of further investigation.

## 3. Discussion

The JAZ gene family has been found in numerous species, including *Arabidopsis* [7], *Solanum lycopersicum* [40], *Hevea brasiliensis* [41], and sunflowers [42], thanks to the enrichment of whole genome sequencing data. However, not much is known about this gene family’s expression and function in TKS, a crop that shows promise as a substitute for producing NR [6]. As a result, our objective was to conduct a genome-wide search for JAZ genes in TKS to elucidate their involvement in the JA signaling pathway and their regulatory role in NR biosynthesis. Based on the 2017 TKS genome data [38], we conducted a comprehensive identification of JAZ proteins in TKS and further investigated their evolutionary relationships, protein structure, gene structure, predicted and studied subcellular locations, protein–protein interactions, and gene expression profiles. This work establishes a foundation for subsequent functional analyses of JAZ genes, aimed at advancing our understanding of JA signaling in TKS. Here, through a local BLAST analysis using the protein sequences of the *Arabidopsis* JAZ family as the query and a database search for the TIFY/JAS domain, 11*TkJAZ* genes were identified in the 2017 TKS genome and the cDNAs were successfully cloned and sequenced (Table 1). After sequencing, these *JAZ* sequences were highly similar to those identified in the 2017 TKS database [38]. BLAST comparison and collinearity analysis revealed that seven of them have counterparts in the recently released 2022 TKS genome, while the remaining four show less than 90% similarity [39]. These results potentially indicate genuine discrepancies between the two TKS genomes from different germplasms or variations in genome assembly and annotation pipelines.

Phylogenetic analysis revealed that the 57 JAZ proteins from four species formed five groups (Figure 1A and Figure 2A). It is worth noting that most *TkJAZs* and *HbJAZs* are in the same subfamily and have close genetic relationships; the only similarity between rubber trees and TKS is that they both can biosynthetically produce NR. Studies have shown that the drainage and de novo biosynthesis of latex is actually a wound response of rubber trees [37]. JA, a master phytohormone, mediates wound responses that have been extensively elucidated in Arabidopsis and numerous other plant species. This phylogenetic relationship suggests that NR biosynthesis in TKS may be linked to the stress response regulated by the JA signaling pathway.

The JA signaling pathway is crucial for plants to respond to both abiotic and biotic stress. Key regulatory factors in this pathway include the alkaline spiral loop transcription factors MYC2, MYC3, MYC4, and MYC5, which initiate the expression of JA-responsive genes [43,44]. Among them, MYC2 is often seen as the central regulators of JA signaling [27]. In this study, protein interaction prediction and *TkJAZs* expression patterns can support these viewpoints. In Figure 3A, it can be observed that *TKJAZs* may interact with *TkCOI1* and *TkMYC2*, *TkMYC3*, and *TkMYC4*, respectively. Therefore, we further analyzed the regulatory relationship of JAZ in the JA signaling pathway using transcriptome data, and the results showed that the expression of *TkCOI1* seemed to be consumptive. After treatment with MeJA, the *TkCOI1* expression level decreased, while the expression level of *TkJAZs* increased. The expression patterns of TkMYC2 and TkJAZs were similar (Figure 3B). These results provide preliminary evidence for the interaction between *TkJAZs* and *TkMYC2* in TKS. To further validate these results, we performed subcellular localization and bimolecular fluorescence complementary analysis on *TkJAZs* and *TkMYC2*.

The results of subcellular localization, Y2H, and BiFC experiments show that both *TkJAZs* and *TkMYC2* are located in the nucleus, and all *TkJAZs* and *TkMYC2* interact with each other in the nucleus (Figure 4, Figure 5 and Figure 6). This indicates that *TkMYC2* is the regulatory target of all *TkJAZs* in TKS, and also demonstrates the crucial role of *TkMYC2* in the JA signaling pathway. Therefore, we will shift the focus of our work to the *TkMYC2* gene.

MYC2 was found to be involved in plant growth and development in rice [45], and some studies have also found that *AtMYC2* can inhibit the growth of the main root and promote the occurrence of lateral roots [46]. Our study found that OE-*TkMYC2*-transgenic TKS roots were longer than wild-type roots (Figure 7). This has a promoting effect on both the main and lateral roots, indicating that overexpression of *TkMYC2* is effective in promoting root elongation in TKS. The reason may be that the MYC2 gene of different species exhibits functional diversity or that JA can regulate latex tube differentiation and latex synthesis [37]. Moreover, gum-producing plants have special latex duct tissue, and *TkMYC2* may regulate the expression of genes related to latex duct cell differentiation, leading to root elongation. However, its molecular mechanism still needs further research.

The genes belonging to the *SRPP/REF* family are crucial for the synthesis of rubber. Rubber trees have been shown to have SRPP protein on the outside and REF protein embedded in the lipid membrane of the rubber particle. To cooperatively control latex synthesis, SRPP proteins can interact with one another and bind to REF protein [47]. The TKS genome contained ten *TkSRPP/REF* family genes that were examined. MeJA-RNA-seq was used to evaluate eight differentially expressed *TkSRPP/REF* family genes (Figure 8A). MeJA induction resulted in the upregulation of three genes (*TkSRPP3, TkSRPP34*, and *TkREF1*) (Figure 8A). According to a study, overexpression of *TkSRPP3* in TKS can lead to an increase in the amount of latex in roots, whereas RNAi-mediated downregulation of *TkSRPP3* expression can decrease this amount and lower the molecular weight of rubber hydrocarbons [34]. Our study found that overexpression of the *TkMYC2* gene can regulate the upregulation of eight *TkSRPP/REF* family genes (Figure 8C).

The aforementioned findings suggest that the JA signaling pathway in TKS may regulate the rubber synthesis route by controlling the expression of the *TkMYC2* and *TkSRPP/REF* family genes; however, additional experiments are necessary to confirm the molecular mechanism involved.

## 4. Materials and Methods

### 4.1. Plant Materials

TKS was collected from the Ili region of the Tekes River basin in Xinjiang, China, and successfully propagated and planted in our laboratory. Subsequently, non-sterile TKS seedlings were cultured in the plant culture room under the following conditions (nutritional soil/vermiculite = 1:1, temperature 25 °C, light 16 h/dark 8 h). In addition, sterile cultivation was carried out after seed disinfection for gene overexpression experiments. Subsequently, plant tissue materials for qRT-PCR were collected in tinfoil (three biological replicates per plant tissue). Subsequently, the tin foil containing plant materials was rapidly frozen using liquid nitrogen and stored in a −80 °C freezer.

### 4.2. Identification and Characterization of JAZ Genes in TKS

After downloading the 13 previously discovered and published JAZ protein sequences in *Arabidopsis* from TAIR (https://www.Arabidopsis.org) [48], queries were used to perform local BLAST algorithm-based searches against 2017 TKS genome databases from CNCB (https://ngdc.cncb.ac.cn/) to identify JAZs from the 2017 TKS genome. Additionally, the TKS genomics database was used to retrieve the protein sequences. Using Tbtools’ Simple HMM search, the proteins containing the TIFY (PF06200) and Jas (PF09425) domains were found [49]. To obtain JAZ candidate protein sequences, we combined these two methods and obtained 11 JAZ candidate proteins. Then, using the NCBI’s CDD tools (http://www.ncbi.nlm.nih.gov/Structure/cdd/wrpsb.cgi/, accessed on 12 March 2023), we analyzed the 11 JAZ candidate protein sequences. The protein sequences were manually selected based on the following criteria: a complete TIFY domain, followed by a complete Jas domain (also known as CCT_2 domain), and no other domains, such as the GATA domain [50]. Finally, all 11 JAZ candidate proteins were identified as JAZ proteins.

Subsequently, we performed motif analysis using the MEME program (http://meme-suite.org/tools/meme accessed on 11 March 2023). Additionally, the length of the TkJAZs sequences was determined using the ExPASy tool [51] (https://web.expasy.org/compute_pi/ accessed on 5 March 2023). Prediction of subcellular localization of TkJAZ proteins was accomplished using Plant-mPLoc (http://www.csbio.sjtu.edu.cn/).

### 4.3. Phylogeny, Gene Structure, and Chromosomal Localization of TkJAZs

To investigate the phylogenetic relationships of TkJAZ proteins, JAZ protein sequences from *Hevea brasiliensis* were obtained from Chao et al.‘s study [41], while rice JAZ protein sequences were sourced from the UniProt database. Multiple sequence alignments (MSAs) of JAZ protein sequences from TKS, rubber tree, rice, and Arabidopsis were conducted using MEGA 7.0, and a phylogenetic tree was constructed with MEGA 7.0 using the neighbor-joining (NJ) method with 1000 bootstrap replications [52]. Analysis of the exon and intron organization of the TkJAZ gene was performed using the Gene Structure Display Server (GSDS, http://gsds.gao-lab.org/) tool, which included examination of intron distribution patterns, phases, and intron–exon boundaries [53]. The collinearity analysis was completed using One-step MCscanX software in Tbtools v2.096 [49], and visualization of conserved motifs, gene structures, and collinearity mapping was carried out using Tbtools.

### 4.4. RNA Isolation and cDNA Synthesis

Total RNAs were collected from TKS roots following the instructions included with the TransGen Biotech Corporation (TransGen Biotech, Beijing, China) general plant total RNA extraction kit. RNA integrity was assessed by electrophoresis on a 2% agarose gel following DNase I digestion to eliminate any remaining gDNA. Reverse transcription was performed using 1 μg of total RNA with the EasyScript^®^ One-Step gDNA Removal and cDNA Synthesis Kit (TransGen Biotech, Beijing, China).

### 4.5. Subcellular Localization, Y2H, and BiFC Analysis of Eleven TkJAZ and TkMYC2 Proteins

To verify the predicted subcellular localization and protein–protein interactions of TkJAZs and TkMYC2 proteins, the full-length coding sequences (CDSs) of all *TkJAZs* and *TkMYC2* genes were successfully cloned and ligated into the T-vector for sequencing. (listed in Table S1). The CDSs of *TkJAZs* and *TkMYC2* were amplified using primers containing homologous arms compatible with the expression vectors (listed in Tables S2 and S3). Subsequently, following the instructions of the ClonExpress^®^ One Step Cloning Kit (Vazyme, Nanjing, China), the sequences were cloned into the *SmaI*-digested pCAMBIA1300-eGFP, pGBKT7, pGADT7, pSPYNE-35s, and pSPYCE-35s vectors by One Step Cloning. (The pSPYNE-35s and pSPYCE-35s vectors contain the potent 35S promoter derived from cauliflower mosaic virus (CaMV 35S), which exhibits strong transcriptional activity in plant cells. This ensures high levels of target protein expression in gene expression experiments.) The one-step cloning method is as follows: The vector is linearized, and the ends of the linearized vector are incorporated into the 5′ ends of the forward and reverse PCR primers. This ensures that the PCR products have sequences at their 5′ and 3′ ends that are homologous to the ends of the linearized vector (15–20 bp). After mixing the PCR product and the linearized vector in a specific ratio, the mixture is incubated at 37 °C for 30 min in the presence of a recombinant enzyme to facilitate targeted cloning.

The PCR products of *TkJAZ1-11* were inserted into pGADT7 to construct prey vectors, while *TkMYC2* was inserted into pGBKT7 to construct bait vectors. The successfully constructed pGADT7-*TkJAZs* and pGBKT7-*TkMYC2* plasmids were transferred to the Y2HGold yeast strain to test the autoactivation of the bait vectors using yeast strains containing pGBKT7-*TkMYC2+*pGADT7 vectors. To assess protein–protein interactions, the corresponding bait and prey vectors (pGBKT7-*TkMYC2+*pGADT7-*TkJAZs*) were co-transformed into Y2HGold. Then, the successfully transformed yeast strains were spotted on the surfaces of solid SD/-Trp/-Leu, SD/-His/-Trp/-Ade/-Leu/, and SD/-His/-Trp/-Ade/-Leu/X-α-Gal medium, and the growth and color development of the colonies were observed.

Subsequently, the successfully constructed plasmid was transferred into Agrobacterium GV3101 using the conventional freeze–thaw method. Next, pCAMBIA1300-*MADS-*mCherry, pCAMBIA1300-*TkMYC2*-GFP, pCAMBIA1300-*TkJAZs*-GFP, pSPYNE-*TkMYC2*, and pSPYNE-*TkJAZs* GV3101 were quickly infiltrated into the strain using an expression buffer (10 mM MES pH 5.6, 10 mM MgCl_2_, 200 µM acetosyringone) into 5-week-old *N. benthamiana* leaves [54]. Among them, pSPYNE-*TkMYC2* GV3101 was needed to co-infiltrate with the different pSPYCE-*TkJAZ* GV3101. After the infiltrated *N. benthamiana* was cultured in the dark for 16 h and then cultivated for 2–3 days in a normal environment (light/dark:16 h/8 h), we took the leaf epidermis and made temporary glass slides. The fluorescence signals were detected by a laser confocal microscope (Nikon AX R, Nikon, Shanghai, China).

Physical maps of vectors are in Figure S3.

### 4.6. Overexpression of TkMYC2 and Expression Analysis of TkSRPP/REF Genes by MeJA-RNA-Seq and qRT-PCR in TKS

The CDS of *TkMYC2* was amplified using primers containing homologous arms compatible with overexpression vectors. (listed in Table S4). Subsequently, following the instructions of the ClonExpress^®^ One Step Cloning Kit (Vazyme, Nanjing, China), the *TkMYC2* gene was cloned into the BamH1 and Pst1-digested pCAMBIA2300-35S vector via one-step cloning (physical map of the pCAMBIA2300-35s vector is seen in Figure S3). Then, the successfully constructed plasmid was transferred into Agrobacterium GV3101 using the conventional freeze–thaw method. Next, we obtained the sterile *TkMYC2* gene overexpressing TKS seedlings using the leaf disk method; the TKS plant tissue culture methods refer to the research of Liang et al. [55]. We screened TKS tissue-cultured seedlings infected with pCAMBIA2300-35s-*TkMYC2, GV3101* using Kan resistance; after the initial screening, the plants were cultured normally for 6 months to collect seeds, which were sown in 1/2 MS medium containing Kan resistance for secondary screening. The plants that survived and developed normally were cultured in nutrient soil for 3 months, and root DNA and root RNA were extracted for PCR (PCR Master Mix Kit: Vazyme, Nanjing, China) and qRT-PCR detection to determine whether the overexpressed genes were stably inherited. DNA-PCR identified TKS candidate transgenic seedlings using 35S upstream primers and target gene downstream primers (listed in Table S4). We identified gene overexpression levels in transgenic TKS using qRT-PCR primers (listed in Table S5).

Primer 5 software was utilized to design primers specific for qRT-PCR (see Table S5). Following the manufacturer’s instructions, the ChamQ Universal SYBR qPCR Master Mix Kit (Vazyme, Nanjing, China) was employed to conduct qRT-PCR on a LightCycler 480TM. The relative gene expression analysis was computed using the 2^−ΔΔCt^ technique, with GAPDH serving as the internal reference gene.

### 4.7. Natural Rubber Extraction

First, we chopped the roots finely and transferred them to a test tube with a capacity of 20 mL. Then, we added 8 mL of a 3% sodium hydroxide solution. After an hour of boiling, we swapped out the test tube for a 3% sodium hydroxide solution and cooked it again for an additional hour. After repeatedly washing the roots in clean water until colorless, we crushed the roots into thin slices to extract the root core from the main root. After adding 8 mL of 4% diluted sulfuric acid, we boiled for a further 8 min. We removed the diluted sulfuric acid liquid, and then neutralized it with 96% anhydrous ethanol. The root residue was then placed in a beaker and dried at 60 °C to a consistent weight [56,57].

### 4.8. Transcriptome Data Analysis

With MeJA-treated transcriptome data from our published studies [58], MeJA treatment was conducted on three-month-old wild seedlings. Nine seedlings of identical growth stages and similar heights were randomly selected for exposure to 0.8 mmol/L MeJA. MeJA was initially dissolved in absolute ethanol and subsequently diluted to the required concentrations with distilled water. Treatments were administered for durations of 6 and 24 h, while control seedlings remained untreated (0 h). Both primary and lateral roots were harvested from both the control and experimental groups, with three biological replicates performed. Collected samples were promptly frozen in liquid nitrogen and stored at −80 °C for subsequent RNA extraction.

## 5. Conclusions

In summary, a total of 11 JAZ family members were identified from the 2017 TKS genome database. Phylogenetic analysis categorized these proteins into two major groups and six minor groups, which was supported by their exon/intron structures, motifs, and domains. All TkJAZ proteins possess conserved TIFY and Jas domains. Subcellular localization results indicated nuclear localization of TkMYC2 and all TkJAZs. Y2H and BiFC assays demonstrated interactions between TkMYC2 and all TkJAZs. Finally, overexpression of *TkMYC2* increases the biomass of TKS roots and promotes the biosynthesis of TKS NR by regulating the expression of the *TkSRPP/REF* gene family.

In this study, the overexpression of the *TkMYC2* gene significantly impacted the phenotype of TKS. Consequently, subsequent experiments should investigate the role of *TkMYC2* in stress resistance and focus on the effect of the *TkSRPP/REF* gene family on NR biosynthesis.

## Figures and Tables

**Table 1:** *TkJAZs* identified in the genomes of TKS.

Gene Name	ID (2017) [38]	Length (aa)	S.L.	ID (2022) [39]	Identity
*TkJAZ1*	evm.model.utg2371.2	263	Nucleus	TkA08G082930.1	100%
*TkJAZ2*	evm.model.utg8854.15	289	Nucleus	TkA01G566490.1	100%
*TkJAZ3*	evm.model.utg11729.12	172	Nucleus	TkA08G341430.1	99%
*TkJAZ4*	evm.model.utg10778.7	103	Nucleus	TkA07G069770.1	100%
*TkJAZ5*	evm.model.utg17755.4	255	Nucleus	TkA03G394100.1	48%
*TkJAZ6*	evm.model.utg8163.14	203	Nucleus	TkA08G068880.1	55%
*TkJAZ7*	evm.model.utg13770.9	288	Nucleus	TkA06G027940.1	49%
*TkJAZ8*	evm.model.utg2308.28	222	Nucleus	TkA01G474510.1	100%
*TkJAZ9*	evm.model.utg3207.6	156	Nucleus	TkA03G585620.1	83%
*TkJAZ10*	evm.model.utg25864.5	226	Nucleus	TkA08G163560.1	100%
*TkJAZ11*	evm.model.utg6907.10	204	Nucleus	TkA08G068880.1	100%

ID (2017): identity code of *TkJAZs* in the 2017 version of a TKS genome; Length (aa): protein amino acid number; S.L.: the probable subcellular location predicted by Plant-mPLoc; ID (2022): identity code of *TkJAZs* in the latest released version of a TKS genome; Identity: identity of the coding regions of *TkJAZs* in the two Tk genome versions.

## Data Availability

We submitted the gene sequences to the National Center for Biotechnology Information (NCBI). The GenBank accession numbers for the nucleotide sequences provided by NCBI are available: *TkMYC2*: MW145492; *TkJAZ1*: PQ015166; *TkJAZ2*: PQ015167; *TkJAZ3*: PQ015168; *TkJAZ4*: PQ015169; *TkJAZ5*: PQ015170; *TkJAZ6*: PQ015171; *TkJAZ7*: PQ015172; *TkJAZ8*: PQ015173; *TkJAZ9*: PQ015174; *TkJAZ10*: PQ015175; *TkJAZ11*: PQ015176; *GAPDH*: PQ015177; *TkSRPP1*: PQ015178; *TkSRPP2*: PQ015179; *TkSRPP4*: PQ015180; *TkSRPP5*: PQ015181; *TkSRPP6*: PQ015182; *TkSRPP7*: PQ015183; *TkSRPP9*: PQ015184; and *TkREF1*: PQ015185. The primers involved in this research are all in the Table S5, and the templates synthesized by the primers are all cDNA. The TKS genome and nucleotide sequences were deposited in the Genome Warehouse (GWH; http://bigd.big.ac.cn/gwh/, accessed on 11 June 2023) under the accession number PRJCA000437.

## Supplementary Materials

The following supporting information can be downloaded at https://www.mdpi.com/article/10.3390/plants13152034/s1: Table S1: T-vector for sequencing; Table S2: Subcellular localization vector construction primers; Table S3: BiFC vector construction primers; Table S4: Overexpression vector construction primers; Table S5: qRT-PCR primers; Figures S1 and S2: Overexpression verification; Figure S3: Physical maps of vectors.

## References

[1] Krotkov G. (1945). A review of literature on *Taraxacum kok-saghyz* Rod. Bot. Rev..

[2] Van Beilen J.B., Poirier Y. (2007). Gunyule and Russian dandelion as alternative sources of natural rubber. Crit. Rev. Biotechnol..

[3] Cornish K., Xie W., Kostyal D.A., Shintani D.K., Hamilton R.G. (2015). Immunological Analysis of the Alternate Rubber Crop *Taraxacum kok-saghyz* Indicates Multiple Proteins Cross-Reactive with *Hevea brasiliensis* Latex Allergens. J. Biotechnol. Biomater..

[4] McAssey E.V., Gudger E.G., Zuellig M.P., Burke J.M. (2016). Population Genetics of the Rubber-Producing Russian Dandelion *Taraxacum kok-saghyz*. PLoS ONE.

[5] Luo Z., Iaffaldano B.J., Zhuang X., Fresnedo-Ramirez J., Cornish K. (2017). Analysis of the first *Taraxacum kok saghyz* transcriptome reveals potential rubber yield related SNPs. Sci. Rep..

[6] Ramirez-Cadavid D.A., Comish K., Michel F.C. (2017). *Taraxacum kok-saghyz* (TK): Compositional analysis of a feedstock for natural rubber and other bioproducts. Ind. Crops Prod..

[7] Liu B., Seong K., Pang S., Song J., Gao H., Wang C., Zhai J., Zhang Y., Gao S., Li X. (2021). Functional specificity, diversity, and redundancy of *Arabidopsis* JAZ family repressors in jasmonate and COI1-regulated growth, development, and defense. New Phytol..

[8] Browse J., Howe G.A. (2008). New weapons and a rapid response against insect attack. Plant Physiol..

[9] De Geyter N., Gholami A., Goormachtig S., Goossens A. (2012). Transcriptional machineries in jasmonate-elicited plant secondary metabolism. Trends Plant Sci..

[10] Farmer E.E., Ryan C.A. (1990). Interplant communication: Airborne methyl jasmonate induces synthesis of proteinase inhibitors in plant leaves. Proc. Natl. Acad. Sci. USA.

[11] Gonzales-Vigil E., Bianchetti C.M., Phillips G.N., Howe G.A. (2011). Adaptive evolution of threonine deaminase in plant defense against insect herbivores. Proc. Natl. Acad. Sci. USA.

[12] Mohan S., Ma P.W.K., Pechan T., Bassford E.R., Williams W.P., Luthe D.S. (2006). Degradation of the *S. frugiperda* peritrophic matrix by an inducible maize cysteine protease. J. Insect Physiol..

[13] Van Loon L.C., Rep M., Pieterse C.M.J. (2006). Significance of inducible defense-related proteins in infected plants. Annu. Rev. Phytopathol..

[14] Dicke M., Baldwin I.T. (2010). The evolutionary context for herbivore-induced plant volatiles: Beyond the ‘cry for help’. Trends Plant Sci..

[15] Hudgins J.W., Christiansen E., Franceschi V.R. (2004). Induction of anatomically based defense responses in stems of diverse conifers by methyl jasmonate: A phylogenetic perspective. Tree Physiol..

[16] Li L., Zhao Y.F., McCaig B.C., Wingerd B.A., Wang J.H., Whalon M.E., Pichersky E., Howe G.A. (2004). The tomato homolog of CORONATINE-INSENSITIVE1 is required for the maternal control of seed maturation, jasmonate-signaled defense responses, and glandular trichome development. Plant Cell.

[17] Peiffer M., Tooker J.F., Luthe D.S., Felton G.W. (2009). Plants on early alert: Glandular trichomes as sensors for insect herbivores. New Phytol..

[18] Qi T., Song S., Ren Q., Wu D., Huang H., Chen Y., Fan M., Peng W., Ren C., Xie D. (2011). The Jasmonate-ZIM-Domain Proteins Interact with the WD-Repeat/bHLH/MYB Complexes to Regulate Jasmonate-Mediated Anthocyanin Accumulation and Trichome Initiation in *Arabidopsis thaliana*. Plant Cell.

[19] Radhika V., Kost C., Mithoefer A., Boland W. (2010). Regulation of extrafloral nectar secretion by jasmonates in lima bean is light dependent. Proc. Natl. Acad. Sci. USA.

[20] Shen J., Zou Z., Xing H., Duan Y., Zhu X., Ma Y., Wang Y., Fang W. (2020). Genome-Wide Analysis Reveals Stress and Hormone Responsive Patterns of JAZ Family Genes in *Camellia sinensis*. Int. J. Mol. Sci..

[21] Ju L., Jing Y., Shi P., Liu J., Chen J., Yan J., Chu J., Chen K.-M., Sun J. (2019). JAZ proteins modulate seed germination through interaction with ABI5 in bread wheat and *Arabidopsis*. New Phytol..

[22] Thireault C., Shyu C., Yoshida Y., St Aubin B., Campos M.L., Howe G.A. (2015). Repression of jasmonate signaling by a non-TIFY JAZ protein in Arabidopsis. Plant J..

[23] Zhou M., Memelink J. (2016). Jasmonate-responsive transcription factors regulating plant secondary metabolism. Biotechnol. Adv..

[24] Ke J., Ma H., Gu X., Thelen A., Brunzelle J.S., Li J., Xu H.E., Melcher K. (2015). Structural basis for recognition of diverse transcriptional repressors by the TOPLESS family of corepressors. Sci. Adv..

[25] Pauwels L., Barbero G.F., Geerinck J., Tilleman S., Grunewald W., Perez A.C., Chico J.M., Vanden Bossche R., Sewell J., Gil E. (2010). NINJA connects the co-repressor TOPLESS to jasmonate signalling. Nature.

[26] Atchley W.R., Terhalle W., Dress A. (1999). Positional dependence, cliques, and predictive motifs in the bHLH protein domain. J. Mol. Evol..

[27] Kazan K., Manners J.M. (2013). MYC2: The Master in Action. Mol. Plant.

[28] Breeze E. (2019). Master MYCs: MYC2, the Jasmonate Signaling “Master Switch”. Plant Cell.

[29] Jung C., Zhao P., Seo J.S., Mitsuda N., Deng S., Chua N.-H. (2015). PLANT U-BOX PROTEIN10 Regulates MYC2 Stability in Arabidopsis. Plant Cell.

[30] Zhou W., Torres J.L.L., Blilou I., Zhang X., Zhai Q., Smant G., Li C., Scheres B. (2019). A Jasmonate signaling network activates root stem cells and promotes regeneration. Mol. Plant-Microbe Interact..

[31] Dai L., Kang G., Li Y., Nie Z., Duan C., Zeng R. (2013). In-depth proteome analysis of the rubber particle of *Hevea brasiliensis* (para rubber tree). Plant Mol. Biol..

[32] Brown D., Feeney M., Ahmadi M., Lonoce C., Sajari R., Di Cola A., Frigerio L. (2017). Subcellular localization and interactions among rubber particle proteins from *Hevea brasiliensis*. J. Exp. Bot..

[33] Wang S., Liu J., Wu Y., You Y., He J., Zhang J., Zhang L., Dong Y. (2016). Micromorphological characterization and label-free quantitation of small rubber particle protein in natural rubber latex. Anal. Biochem..

[34] Collins-Silva J., Nural A.T., Skaggs A., Scott D., Hathwaik U., Woolsey R., Schegg K., McMahan C., Whalen M., Cornish K. (2012). Altered levels of the *Taraxacum kok-saghyz* (Russian dandelion) small rubber particle protein, TkSRPP3, result in qualitative and quantitative changes in rubber metabolism. Phytochemistry.

[35] Cherian S., Ryu S.B., Cornish K. (2019). Natural rubber biosynthesis in plants, the rubber transferase complex, and metabolic engineering progress and prospects. Plant Biotechnol. J..

[36] Hillebrand A., Post J.J., Wurbs D., Wahler D., Lenders M., Krzyzanek V., Pruefer D., Gronover C.S. (2012). Down-Regulation of Small Rubber Particle Protein Expression Affects Integrity of Rubber Particles and Rubber Content in *Taraxacum brevicorniculatum*. PLoS ONE.

[37] Deng X., Guo D., Yang S., Shi M., Chao J., Li H., Peng S., Tian W. (2018). Jasmonate signalling in the regulation of rubber biosynthesis in laticifer cells of rubber tree, *Hevea brasiliensis*. J. Exp. Bot..

[38] Lin T., Xu X., Ruan J., Liu S., Wu S., Shao X., Wang X., Gan L., Qin B., Yang Y. (2018). Genome analysis of *Taraxacum kok-saghyz* Rodin provides new insights into rubber biosynthesis. Natl. Sci. Rev..

[39] Lin T., Xu X., Du H., Fan X., Chen Q., Hai C., Zhou Z., Su X., Kou L., Gao Q. (2022). Extensive sequence divergence between the reference genomes of *Taraxacum kok-saghyz* and *Taraxacum mongolicum*. Sci. China-Life Sci..

[40] Chini A., Ben-Romdhane W., Hassairi A., Aboul-Soud M.A.M. (2017). Identification of TIFY/JAZ family genes in *Solanum lycopersicum* and their regulation in response to abiotic stresses. PLoS ONE.

[41] Chao J., Zhao Y., Jin J., Wu S., Deng X., Chen Y., Tian W.-M. (2019). Genome-Wide Identification and Characterization of the JAZ Gene Family in Rubber Tree (*Hevea brasiliensis*). Front. Genet..

[42] Song H., Fu X., Li J., Niu T., Shen J., Wang X., Li Y., Hou Q., Liu A. (2022). Phylogenetic analysis and expression profiles of jasmonate ZIM-domain gene family provide insight into abiotic stress resistance in sunflower. Front. Plant Sci..

[43] Goossens J., Mertens J., Goossens A. (2017). Role and functioning of bHLH transcription factors in jasmonate signalling. J. Exp. Bot..

[44] Boter M., Ruíz-Rivero O., Abdeen A., Prat S. (2004). Conserved MYC transcription factors play a key role in jasmonate signaling both in tomato and *Arabidopsis*. Genes Dev..

[45] Giri M.K., Gautam J.K., Prasad V.B.R., Chattopadhyay S., Nandi A.K. (2017). Rice MYC2 (OsMYC2) modulates light-dependent seedling phenotype, disease defence but not ABA signalling. J. Biosci..

[46] Chen Y.C., Wong C.L., Muzzi F., Vlaardingerbroek I., Kidd B.N., Schenk P.M. (2014). Root defense analysis against *Fusarium oxysporum* reveals new regulators to confer resistance. Sci. Rep..

[47] Yamashita S., Yamaguchi H., Waki T., Aoki Y., Mizuno M., Yanbe F., Ishii T., Funaki A., Tozawa Y., Miyagi-Inoue Y. (2016). Identification and reconstitution of the rubber biosynthetic machinery on rubber particles from *Hevea brasiliensis*. eLife.

[48] Lamesch P., Berardini T.Z., Li D., Swarbreck D., Wilks C., Sasidharan R., Muller R., Dreher K., Alexander D.L., Garcia-Hernandez M. (2012). The Arabidopsis Information Resource (TAIR): Improved gene annotation and new tools. Nucleic Acids Res..

[49] Chen C., Chen H., Zhang Y., Thomas H.R., Frank M.H., He Y., Xia R. (2020). TBtools: An Integrative Toolkit Developed for Interactive Analyses of Big Biological Data. Mol. Plant.

[50] Zhang Y., Gao M., Singer S.D., Fei Z., Wang H., Wang X. (2012). Genome-wide identification and analysis of the TIFY gene family in grape. PLoS ONE.

[51] Gasteiger E., Gattiker A., Hoogland C., Ivanyi I., Appel R.D., Bairoch A. (2003). ExPASy: The proteomics server for in-depth protein knowledge and analysis. Nucleic Acids Res..

[52] Kumar S., Stecher G., Tamura K. (2016). MEGA7: Molecular Evolutionary Genetics Analysis Version 7.0 for Bigger Datasets. Mol. Biol. Evol..

[53] Hu B., Jin J., Guo A.-Y., Zhang H., Luo J., Gao G. (2015). GSDS 2.0: An upgraded gene feature visualization server. Bioinformatics.

[54] Sparkes I.A., Runions J., Kearns A., Hawes C. (2006). Rapid, transient expression of fluorescent fusion proteins in tobacco plants and generation of stably transformed plants. Nat. Protoc..

[55] Liang C., Yan Y., Tan Y., Yang X., Cao J., Tang C., Liu K. (2023). Identification of miRNAs and their targets in two *Taraxacum* species with contrasting rubber-producing ability. Front. Plant Sci..

[56] Li Z., Cheng B. (1954). Determination of Rubber Content in *Taraxacum kok-saghyz* by Alkali Boiling Method—Part I: Xinjiang Perennial Grass Roots. Chem. World.

[57] Lin Z. (1954). Determination of Rubber Content in *Taraxacum kok-saghyz* by Alkali Boiling Method (Continued)—Part II: Fresh Grass Roots of Year One or Two. Chem. World.

[58] Cao X., Yan J., Lei J., Li J., Zhu J., Zhang H. (2017). De novo Transcriptome Sequencing of MeJA-Induced *Taraxacum kok-saghyz* Rodin to Identify Genes Related to Rubber Formation. Sci. Rep..

