# Supplementary material for: TkJAZs-TkMYC2-TkSRPP/REF Regulates the Biosynthesis of Natural Rubber in Taraxacum kok-saghyz
Source: Plants (Basel). 2024 Jul 24;13(15):2034. doi: 10.3390/plants13152034 (PMC11314035; doi:10.3390/plants13152034)
Supplement: Supplementary file 1 [file plants-13-02034-s001.zip › plants-3099285-supplementary/Supplementary table and figure/Figure S1S2.pdf]

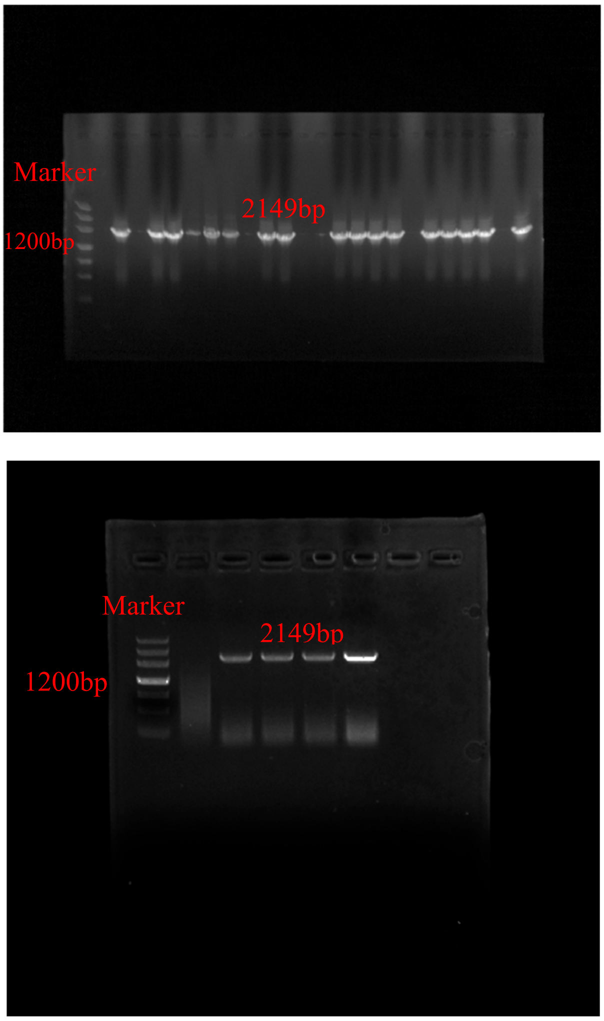


**Figure S1.** Identification of overexpressed plants by DNA PCR. Marker use DNA Marker III: Marker III consists of six DNA fragments of specific sizes: 200 bp, 800 bp, 1200 bp, 2000 bp, 3000 bp, and 4500 bp. Manufacturer:(Vazyme, Nanjing, China)

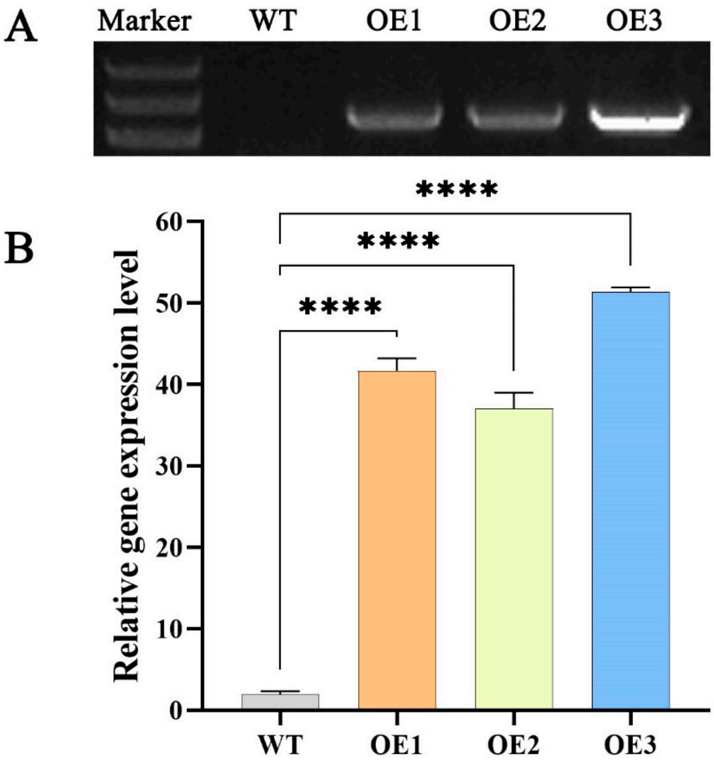


**Figure S2.** Identification of *TkMYC2* overexpressed transgenic *T. kok-saghyz*. (A) Three *TkMYC2* overexpressed transgenic *T. kok-saghyz* lines (OE1, OE2, and OE3) were identified at the DNA level by PCR, performed using 35S forward primer and gene reverse primer. (B) These transgenic *T. kok-saghyz* lines were further verified at the mRNA level by qRT-PCR. Values represent means of n=3±SD (****P<0.0001) from three independent biological experiments.
