# Supplementary material for: TkJAZs-TkMYC2-TkSRPP/REF Regulates the Biosynthesis of Natural Rubber in Taraxacum kok-saghyz
Source: Plants (Basel). 2024 Jul 24;13(15):2034. doi: 10.3390/plants13152034 (PMC11314035; doi:10.3390/plants13152034)
Supplement: Supplementary file 1 [file plants-13-02034-s001.zip › plants-3099285-supplementary/Supplementary table and figure/Figure S3.pdf]

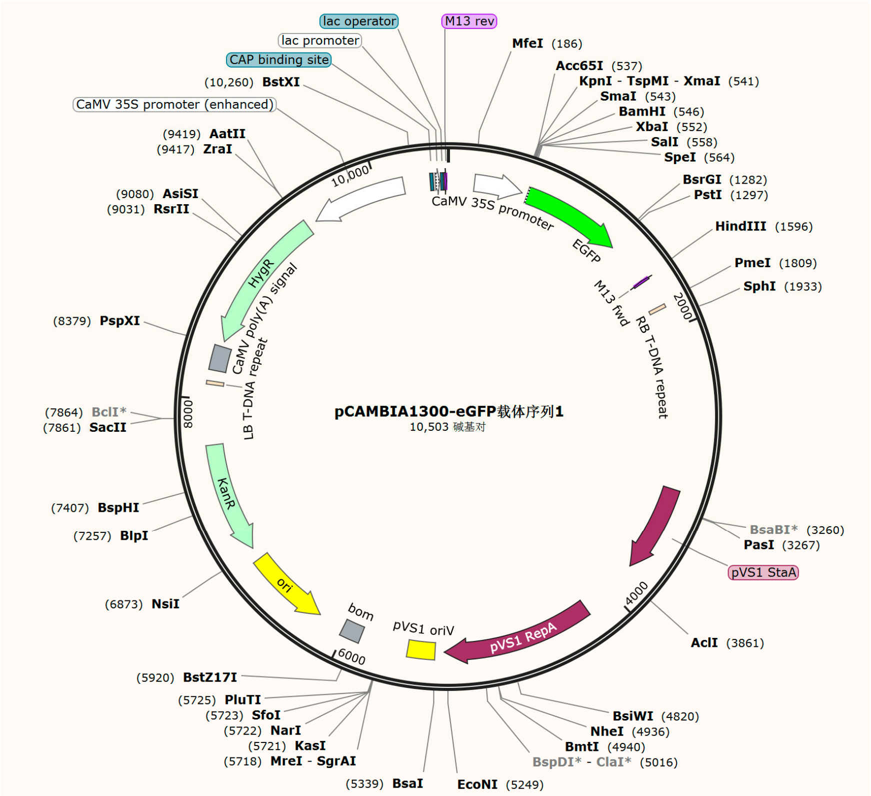

Figure S3-1. physical map of pCAMBIA1300-eGFP vector: Used for subcellular localization experiments.

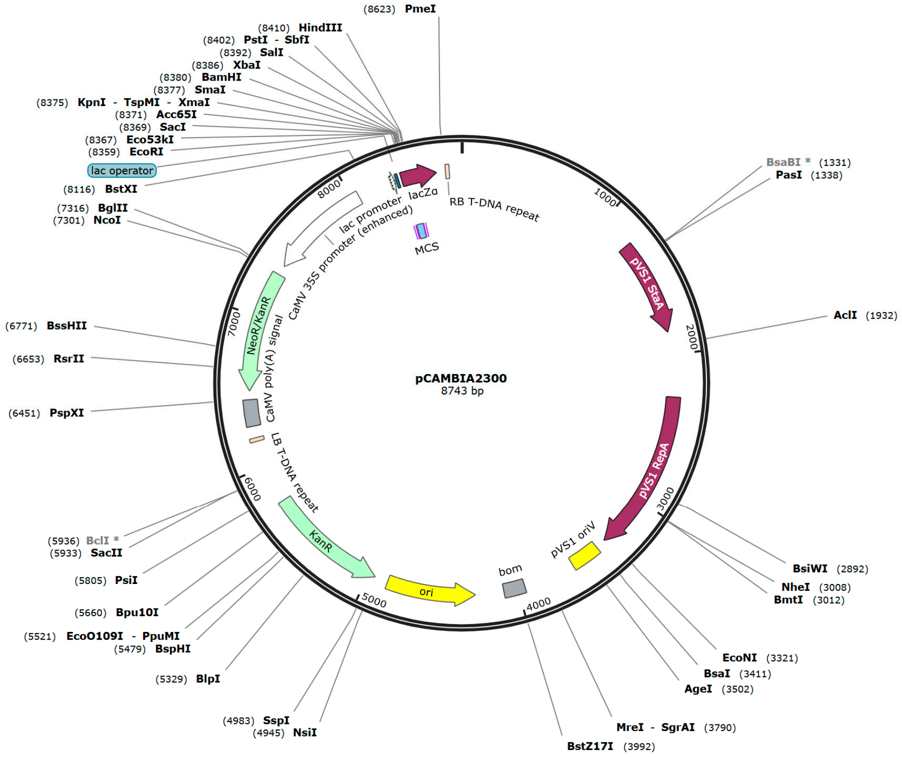

Figure S3-2. physical map of pCAMBIA2300 vector: Used for *TkMYC2* gene overexpression experiments.

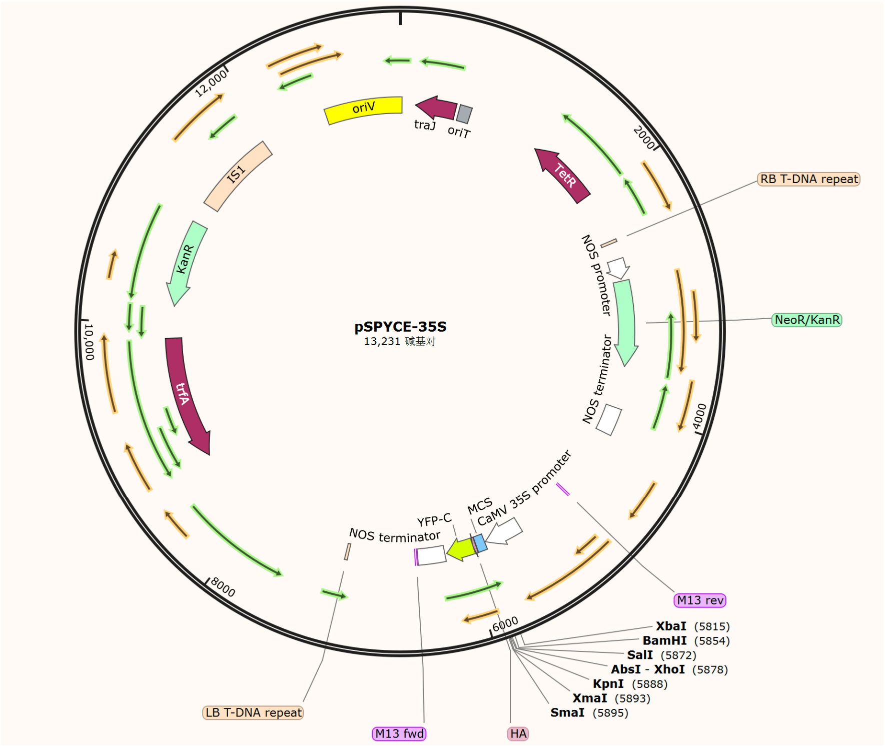

Figure S3-3. Physical map of pSPYCE-35s vector: Expression of TkJAZs-YFP-C protein in BIFC experiments.

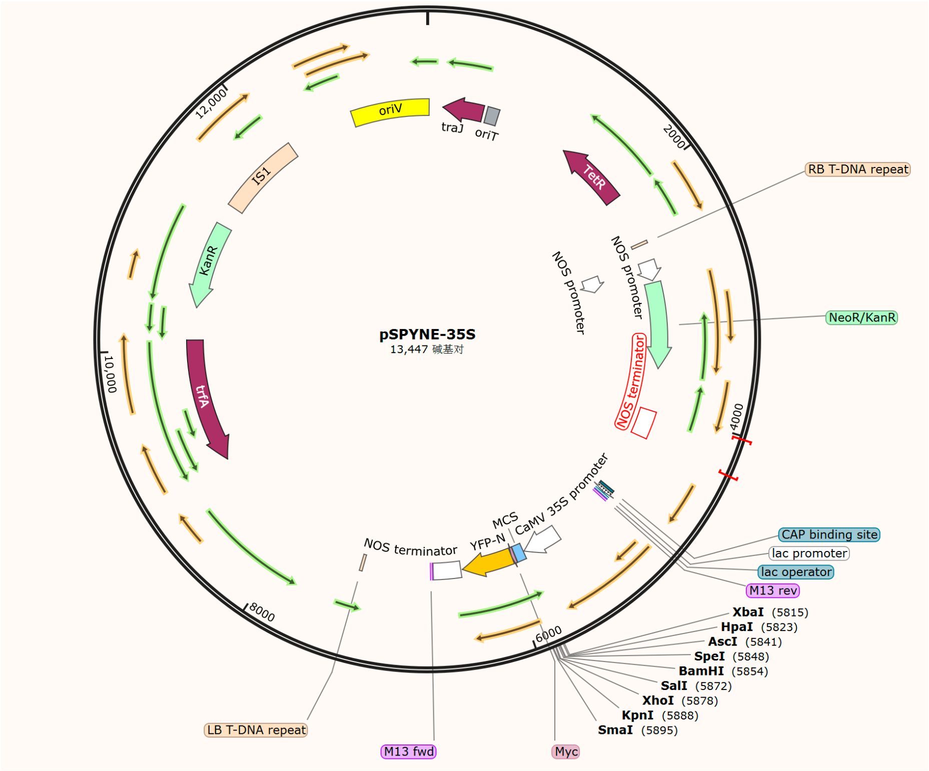

Figure S3-4. Physical map of pSPYNE-35s vector: Expression of TkMYC2-YFP-N protein in BIFC experiments

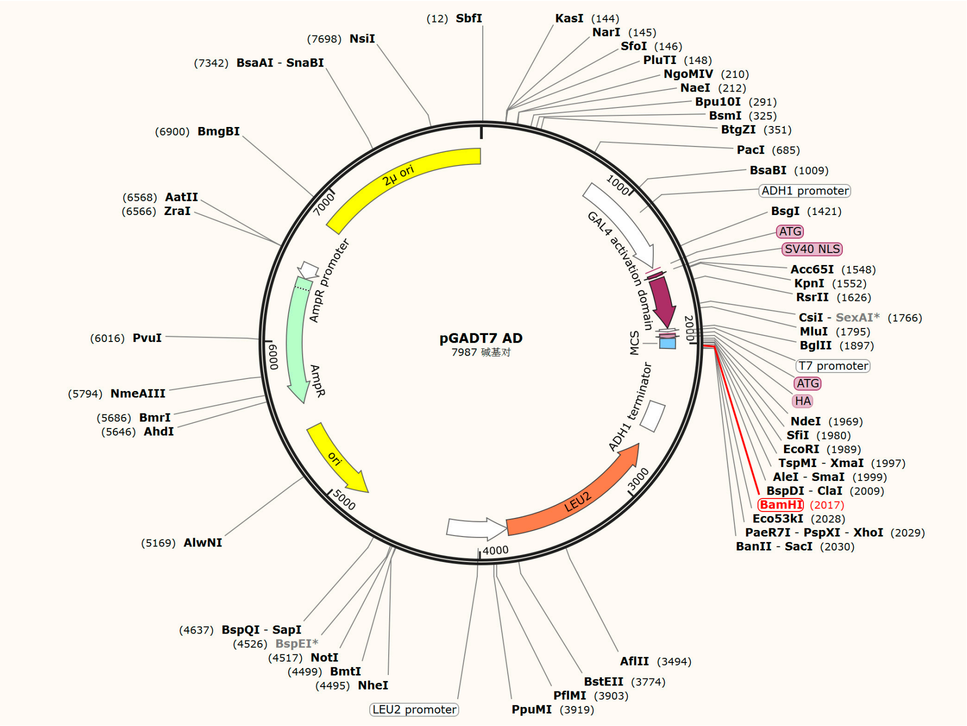

Figure S3-5. Physical map of pGADT7 vector: Construction of pGADT7-TkJAZs prey vector for Y2H experiment

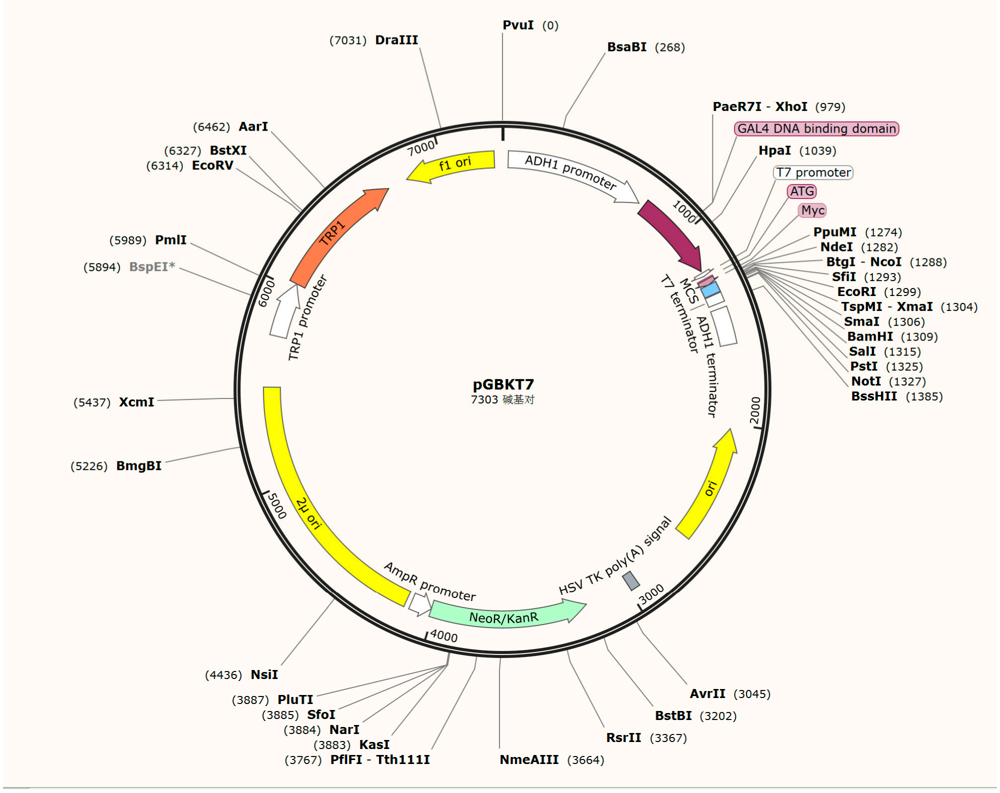

Figure S3-6. Physical map of pGBKT7 vector: Construction of pGBKT7-TkMYC2 bait vector for Y2H experiment
